# Betaine Dietary Supplementation: Healthy Aspects in Human and Animal Nutrition

**DOI:** 10.3390/antiox14070771

**Published:** 2025-06-23

**Authors:** Giovanni Buonaiuto, Alessia Federiconi, Carla Giuditta Vecchiato, Elisa Benini, Attilio Luigi Mordenti

**Affiliations:** 1 Dipartimento di Scienze Mediche Veterinarie (DIMEVET), Alma Mater Studiorum-Università di Bologna, Via Tolara di Sopra 50, 40064 Ozzano Emilia, BO, Italy; giovanni.buonaiuto@unibo.it (G.B.); alessia.federiconi3@unibo.it (A.F.); carla.vecchiato2@unibo.it (C.G.V.); 2 Dipartimento di Scienze Mediche Veterinarie (DIMEVET), Alma Mater Studiorum-Università di Bologna-Unità Operativa di Sede (Cesenatico), Viale Vespucci 2, 47042 Cesenatico, FC, Italy; elisa.benini7@unibo.it

**Keywords:** betaine dietary supplementation, human nutrition, animal nutrition, antioxidant activity, nutraceutical activity

## Abstract

Betaine, a naturally occurring compound primarily derived from sugar beet by-products, has attracted increasing attention for its multifaceted roles in human and animal nutrition. Acting as both an osmolyte and a methyl group donor, betaine contributes to cellular hydration, methylation balance, antioxidant defense, and metabolic regulation. This review provides a comprehensive overview of betaine’s biological functions and its health-promoting effects across species. In humans, betaine supports hepatic function, cardiovascular health, renal protection, and physical performance, mainly by modulating homocysteine metabolism, lipid profiles, and oxidative stress. In animal production systems, it enhances growth, feed efficiency, reproductive performance, and resilience to heat stress, with species-specific applications in monogastrics, ruminants, aquaculture species, and companion animals. The review also explores the molecular mechanisms underlying betaine’s effects, including epigenetic regulation and mitochondrial function, and presents updated evidence on its biosynthesis, bioavailability, and nutrient interactions. Furthermore, the use of betaine derived from agro-industrial by-products aligns with the principles of the circular economy, promoting the sustainable reuse of valuable compounds within the agri-food chain. Despite promising findings, further research is needed to standardize effective dosages and clarify species-specific responses under different physiological and environmental conditions. Overall, betaine emerges as a promising and sustainable functional ingredient with wide-ranging applications in nutrition and health.

## 1. Introduction

Betaine, or N-trimethylglycine (CH_3_)_3_N⁺CH_2_COO^−^, is a naturally occurring zwitterionic compound derived from the amino acid glycine. First identified in the 19th century in sugar beet (*Beta vulgaris*) juice, it is ubiquitously distributed across plants, animals, and microorganisms. Beyond its widespread biological presence, betaine is also used industrially as a solubilizing agent and humectant [1]. Endogenously, betaine is synthesized through the irreversible oxidation of choline and is abundant in several dietary sources. It is most concentrated in plant-derived foods, particularly sugar beet and cereal grains [2]. However, certain seafoods—especially bivalve mollusks such as mussels, oysters, clams, and scallops—also contain appreciable amounts of betaine [2]. Functionally, betaine performs two major physiological roles: as an organic osmolyte, it contributes to cellular hydration and protein stabilization under hyperosmotic stress [3]; and as a methyl donor in one-carbon metabolism, it participates in the remethylation of homocysteine to methionine (Met), catalyzed by betaine-homocysteine methyltransferase (BHMT) [4]. Emerging evidence highlights its metabolic importance in sustaining methylation capacity, modulating lipid and amino acid metabolism, and regulating gene expression via epigenetic pathways. These functions are closely linked to redox homeostasis, as both methylation potential and osmotic regulation influence mitochondrial activity and antioxidant defenses. Moreover, betaine has been implicated in modulating redox-sensitive transcription factors, notably Nrf2, with downstream effects on glutathione synthesis, lipid peroxidation, and inflammatory signaling. In human nutrition, betaine supplementation has been investigated for its efficacy in lowering plasma homocysteine levels, improving hepatic lipid metabolism, and enhancing physical performance. In animal nutrition, dietary inclusion of betaine has been associated with improved growth rates, feed efficiency, carcass composition, thermotolerance, and oxidative status—particularly under environmental or physiological stress conditions.

This review provides an in-depth examination of betaine’s chemical structure, metabolic functions, and health-related effects, with an emphasis on its role in oxidative balance and nutritional physiology. Specific attention is given to its applications in human health and animal production systems, including ruminants, monogastric species, companion animals, and aquaculture. The discussion highlights species-specific responses to dietary supplementation, implications for production sustainability, and recent advancements in betaine sourcing and formulation strategies.

## 2. Industrial Extraction of Betaine from Sugar Beet

The industrial extraction of betaine is intrinsically linked to the evolution of sugar production from *Beta vulgaris*, a process that began approximately two hundred years ago and has since undergone significant technological advancements. Historically, calco-carbonic purification has been the cornerstone of sugar beet processing, involving sequential liming and carbonation steps to purify raw juice. This multi-stage process—comprising lime addition, CO_2_ carbonation, solid-liquid filtration, and ion-exchange resin treatment—effectively removes suspended solids, color bodies, and non-sugar impurities, thereby enhancing the purity of the final sugar product. However, this traditional method presents notable drawbacks, including excessive lime consumption, the generation of calcium-rich sludge, high energy demands, and environmental concerns related to CO_2_ emissions and waste disposal [5]. Furthermore, the harsh calcareous conditions and elevated processing temperatures can degrade or partially destroy thermolabile compounds such as betaine, limiting its recovery from the molasses stream. In response, both industry and research have pursued eco-friendly, lime-free alternatives aimed at preserving valuable co-products while reducing resource consumption and environmental impact [6]. One such innovation involves the direct concentration and crystallization of raw juice, bypassing lime treatment altogether. This approach not only reduces color formation and sugar degradation but also improves crystallization kinetics and product yields.

Simultaneously, membrane filtration techniques—particularly microfiltration—have been adopted to remove suspended solids and colloidal impurities, thereby simplifying the juice matrix prior to further refining. Chromatographic separation, either in batch mode or via simulated moving bed (SMB) systems, has emerged as a powerful addition to the processing toolkit. These methods enable selective partitioning of betaine-containing fractions. SMB systems, in particular, facilitate continuous or semi-continuous processing where eluent streams are recycled within the system, reducing fresh solvent consumption while increasing throughput and betaine purity [7,8,9]. The combination of chromatographic separation with low-temperature crystallization further enhances sugar quality: slower crystal growth at reduced thermal stress produces high-purity sugar while preserving thermolabile co-products. Quantitatively, chromatographic systems have demonstrated betaine removal efficiencies exceeding 96%, representing a significant improvement over traditional purification methods [7,10,11]. These technologies offer precise selectivity, operate under mild conditions, and avoid the use of strong alkaline or inorganic reagents. Energy savings result not only from milder operating conditions and lower thermal requirements for cooling crystallization but also from reduced chemical consumption and minimized waste streams.

However, the adoption of membrane-based and chromatographic technologies requires higher capital investment and specialized operational expertise, which may limit their implementation in legacy plants. Nevertheless, economies of scale, regulatory incentives promoting environmental sustainability, and potential revenues from betaine valorisation increase the attractiveness of these innovative approaches [7,8,9]. By recovering high-purity betaine alongside sugar, sugar beet processing plants can evolve toward integrated biorefineries, aligning with circular economy principles and diversifying their product portfolios.

## 3. Betaine: Chemical Characteristics and Nutritional Properties/Applications

Betaine is considered an important compound due to its role as an organic osmolyte, its nutritional properties, and its ability to donate methyl groups. Betaine can be obtained through dietary sources or synthesized de novo within tissues [11]. In animals, betaine is primarily synthesized in the liver and kidneys via the oxidation of choline. This process involves a two-step enzymatic reaction: choline is first converted to betaine aldehyde by choline oxidase in the mitochondria, followed by oxidation to betaine by betaine aldehyde dehydrogenase [1,3,12].

In plants, betaine is synthesized through two pathways—one involving choline and another involving glycine—occurring in both the chloroplasts and cytosol. One of betaine’s key functions is its role as an osmolyte, which contributes to heat stress tolerance. By stabilizing cellular structures under stress, betaine activates calcium-dependent protein kinases and mitogen-activated protein kinases, which in turn activate stress-responsive transcription factors [13]. This signaling cascade enhances antioxidant enzyme activity, helping to mitigate the damaging effects of reactive oxygen species (ROS) during heat stress. These adaptations enable plants to survive under varying climatic conditions and improve water-use efficiency [14].

In microorganisms, betaine is also synthesized via a two-step choline oxidation process; however, some species additionally employ a three-step methylation of glycine, a pathway exclusive to certain microorganisms [15]. Beyond osmolyte function, betaine serves as a methyl donor in the remethylation of homocysteine to methionine within the betaine-homocysteine methyltransferase pathway. This process influences the concentration of S-adenosylmethionine, thereby affecting DNA methylation of cytosine bases, gene transcription, genomic imprinting, and stability. As a result, betaine supplementation can impact gene silencing and alter gene expression. Notably, betaine is more efficient than phosphatidylcholine in homocysteine remethylation, as phosphatidylcholine must first be metabolized to betaine before contributing to this process. As an osmolyte, betaine regulates cell volume, stabilizes proteins, and mitigates the denaturing effects of urea [2,16]. While betaine is not considered an essential nutrient—since it can be synthesized endogenously—it is necessary to meet daily requirements due to insufficient endogenous production. Betaine’s functional applications extend beyond nutrition. It improves osmotic tolerance in cells by modulating osmolarity, protects microbial strains during storage and recovery, promotes cell growth, and safeguards intracellular components, including enzymes, from stress [15].

## 4. Betaine in Human Nutrition

Betaine is a nutritional component of the human diet, naturally present in a variety of foods and also available as a dietary supplement in cases of deficiency. It is considered a derivative of choline [17,18,19]. As previously described, betaine is utilized by higher organisms due to its unique chemical structure, which confers two primary physiological functions. First, it acts as an osmolyte, protecting cells under stress by promoting water retention, replacing inorganic salts and shielding intracellular enzymes from osmotic or thermal damage [1,3,4]. Second, it serves as a methyl group donor, playing a central role in restoring methionine homeostasis through trans-methylation reactions that support multiple biochemical pathways [1,3,4]. These two functions make betaine essential for maintaining the health of several organs, including the heart, liver and kidneys.

The daily intake of betaine in Western populations depends on both dietary sources and endogenous synthesis. Although the commonly referenced value is the adequate intake of choline (550 mg/d for men [20]), reported values for actual betaine intake vary widely in the literature. For example, Arumugam et al. [1] and Hoffman et al. [21] report a range of 1000–2500 mg/day, whereas Craig [3] and Ashtary-Larky et al. [22] mention much higher intakes of 9000–15,000 mg/day (with an average of approximately 12,000 mg/day). In contrast, other studies by Willingham et al. [4], Moro et al. [23] and Cho et al. [24] report substantially lower intakes, ranging from 100 to 400 mg/day (mean: 208 ± 90 mg/day). However, based on hematological studies in rats, even an intake of 12,000 mg/day may be considered safe in humans [25,26].

Betaine is efficiently absorbed in the small intestine. Initially, it accumulates in enterocytes via active transport mechanisms involving sodium or chloride-coupled amino acid transport systems, and it may also be absorbed through passive, sodium-independent pathways [3,4,5]. Due to its methylated structure, betaine transport is further mediated by the membrane-bound betaine/γ-aminobutyric acid transporter 1 (BGT-1) and amino acid transport system A, particularly under hypertonic stress [1,3,4,27,28,29]. After ingestion, betaine is rapidly absorbed in the duodenum [30,31]. Within the body, it is catabolized to dimethylglycine, which is further degraded to sarcosine and ultimately converted to glycine as part of the methionine cycle. These transformations mainly occur in the mitochondria of liver and kidney cells [1,3,4,31,32].

### 4.1. Betaine and Hepatic Function

Betaine plays a critical role in hepatic physiology, primarily as a methyl donor in one-carbon metabolism and due to its lipotropic properties. It is increasingly recognized for its hepatoprotective effects in both alcoholic liver disease (ALD) and metabolism-associated fatty liver disease (MAFLD) [1,3,33]. Despite differing etiologies—chronic alcohol intake and metabolic dysfunction, respectively—ALD and MAFLD share pathological features including hepatic steatosis, inflammation, fibrosis, and progression to cirrhosis or hepatocellular carcinoma. Hepatic steatosis, the initial stage of both ALD and MAFLD, is characterized by excessive lipid accumulation in hepatocytes [33,34,35]. In both conditions, hepatic steatosis marks the earliest stage and is driven by increased free fatty acid (FFA) influx from adipose tissue, elevated de novo lipogenesis, impaired mitochondrial β-oxidation, and reduced very-low-density lipoprotein (VLDL) secretion [33,34,35]. As a methyl donor, betaine supports methionine regeneration via betaine-homocysteine methyltransferase (BHMT), thereby restoring the hepatic S-adenosylmethionine (SAM): S-adenosylhomocysteine (SAH) ratio—crucial for methylation capacity and epigenetic regulation.

Chronic ethanol exposure disrupts this balance, impairing methylation reactions and phosphatidylcholine synthesis via phosphatidylethanolamine N-methyltransferase (PEMT), thereby compromising VLDL secretion [11]. Betaine supplementation restores PEMT activity, enhances VLDL export, and reduces hepatic triglyceride accumulation. Moreover, betaine attenuates oxidative stress and inflammation by enhancing antioxidant capacity and downregulating cytokines such as TNF-α and COX-2 [36]. In animal models of alcohol-induced liver injury, betaine supplementation reduced endoplasmic reticulum stress, improved mitochondrial ultrastructure, and reversed ethanol-induced hypomethylation of genes involved in nitric oxide synthesis [11]. Betaine has also been shown to prevent the methyl-folate trap caused by chronic alcohol intake, reduce vitamin A depletion, and preserve erythrocyte membrane integrity. In the context of MAFLD, betaine offers additional benefits beyond hepatic lipid metabolism. It strengthens the intestinal barrier by upregulating tight junction proteins and modulates gut microbiota composition, thereby reducing endotoxemia and hepatic inflammation [11]. Betaine also improves adipose tissue function by restoring normal adipokine profiles and enhancing mitochondrial activity.

The liver is the primary site of ethanol metabolism, which occurs via three main enzymatic pathways: (i) alcohol dehydrogenase (ADH), converting ethanol to acetaldehyde using NAD+; (ii) the microsomal ethanol-oxidizing system (MEOS), primarily involving cytochrome P450 2E1; and (iii) catalase-mediated oxidation in peroxisomes [1,37]. Acetaldehyde, a toxic metabolite, is further oxidized to acetate by mitochondrial aldehyde dehydrogenase (ALDH) [1,38,39]. Ethanol metabolism impairs the methionine cycle and increases homocysteine levels, leading to reduced methylation potential. Betaine restores the SAM:SAH ratio and supports hepatic methylation reactions, thereby reducing triglyceride accumulation and liver injury [40,41,42].

Moreover, betaine counters oxidative stress and inflammation induced by chronic alcohol intake by boosting antioxidant defenses and repressing proinflammatory cytokines. It also reverses epigenetic changes such as the hypomethylation of genes like nitric oxide synthase (NOS), thus preserving normal gene expression [1,43,44]. MAFLD, formerly known as non-alcoholic fatty liver disease (NAFLD), shares several pathophysiological mechanisms with ALD but is mainly driven by metabolic imbalances, including excess caloric intake, insulin resistance, and altered adipose tissue function. The disease progresses from simple steatosis to steatohepatitis, fibrosis, and hepatocellular carcinoma [45,46,47]. Betaine supplementation exerts protective effects by improving intestinal barrier integrity, modulating gut microbiota, reducing systemic inflammation, and enhancing mitochondrial and adipose tissue function [1,48,49].

### 4.2. Cardiovascular and Metabolic Benefits of Betaine Supplementation

The combination of betaine and guanidinoacetate (glycocyamine) has been shown to alleviate symptoms in individuals with chronic diseases, including cardiovascular disorders, without evidence of toxicity. Clinical observations report improvements in general well-being, reductions in fatigue, enhanced muscular strength and endurance, and improvements in both physical and cognitive performance [1,50,51]. In patients with cardiovascular conditions such as cardiac decompensation and congestive heart failure, betaine supplementation has been associated with improved cardiac function [52]. Additional reported benefits include weight gain in undernourished individuals, attenuation of symptoms in arthritis and asthma, and enhanced libido [3,51,53,54].

Betaine has also demonstrated transient antihypertensive effects and improved glucose tolerance in both diabetic and non-diabetic individuals [55]. Moreover, several studies suggest that betaine positively modulates lipid metabolism and may contribute to the prevention of atherosclerosis [9,56,57,58]. Notably, higher dietary intake of betaine has been associated with reduced cardiovascular-related mortality in individuals with atherosclerotic disease [9,56,57,58,59].

Hyperhomocysteinemia, caused by disruptions in the methionine cycle due to genetic polymorphisms or nutritional deficiencies, is strongly associated with an increased risk of cardiovascular disease, stroke, Alzheimer’s disease, and other metabolic disorders [3,33,58,60,61,62]. Betaine acts as a methyl donor in the remethylation of homocysteine to methionine via BHMT, thereby reducing plasma homocysteine levels and mitigating related health risks. Clinical studies have confirmed that betaine supplementation effectively lowers homocysteine concentrations in individuals with pre-existing cardiovascular disease, while epidemiological data show an inverse relationship between dietary intake of betaine-rich foods and cardiovascular risk in healthy populations [3,33,63,64,65].

Mild hyperhomocysteinemia—a common metabolic condition characterized by moderately elevated homocysteine levels—represents a significant contributor to cardiovascular risk. It is commonly managed using a combination of betaine, folic acid, and vitamin B6 [10,33,66,67]. However, betaine alone has also been shown to prevent increases in homocysteine concentrations and support cardiovascular health, particularly in individuals with impaired methylation capacity [68]. Furthermore, genetic polymorphisms affecting homocysteine metabolism may influence the individual response to betaine supplementation and modulate susceptibility to cardiovascular disease. In such cases, betaine supplementation may serve as an effective therapeutic intervention to restore metabolic balance [69].

Importantly, the dosage of betaine plays a critical role in ensuring cardiovascular safety. According to Ashtary-Larky et al. [70], daily doses of 4 g or more may adversely affect the lipid profile, particularly in individuals with underlying metabolic disorders. In contrast, supplementation with lower doses (<4 g/day) appears to offer comparable reductions in plasma homocysteine without negatively impacting blood lipid levels [70]. These findings suggest that a cardiovascular-safe range for betaine intake is likely below 4 g/day. Therefore, the prescribed dosage of betaine should be carefully tailored to the individual’s health status, balancing therapeutic efficacy with metabolic safety.

### 4.3. Kidney Function and Osmoregulation

BHMT is abundantly expressed in the kidneys of primates and pigs, whereas it is generally absent in the organs of other monogastric species. Within the kidney, BHMT expression is restricted to the proximal tubules of the renal cortex and is not detected in the medulla. The enzyme is localized in the cytosol of both the liver and kidney, and its activity is regulated by extracellular tonicity [3,71]. Under hypertonic conditions, BHMT activity is downregulated to preserve intracellular betaine for its role as an osmoprotectant. Conversely, in hypotonic environments, BHMT expression is upregulated to support the regulation of cellular turgor pressure. In addition to its osmoregulatory functions, betaine has demonstrated nephroprotective effects. In patients with renal disease receiving folic acid supplementation, betaine has been shown to reduce plasma homocysteine levels following methionine loading, although it does not significantly affect fasting homocysteine concentrations [72,73].

### 4.4. Performance, Body Mass Composition, Heat Tolerance and General Well-Being

Animal studies have demonstrated that betaine supplementation can positively influence body composition by reducing fat mass. However, findings from human studies remain inconclusive and, in some cases, contradictory [22,74]. Betaine plays a key role in creatine biosynthesis and may contribute to improved muscle recovery, endurance, and strength. Dietary supplementation with a combination of betaine and guanidinoacetate has shown clinical benefits in patients with poliomyelitis, although limited efficacy has been observed in motor neuron diseases; nonetheless, psychological improvements have been reported [74,75]. Moreover, betaine has been proposed as an ergogenic aid capable of enhancing athletic performance by improving energy metabolism and delaying fatigue, particularly when administered in combination with electrolyte solutions. In addition to its potential ergogenic properties, betaine supports the physiological function of the liver, heart, and kidneys, modulates plasma homocysteine concentrations, and may regulate gene expression through epigenetic mechanisms [75,76,77].

## 5. Betaine in Monogastrics Nutrition

### 5.1. Swine

Betaine is widely used in pig nutrition due to its dual role as a methyl group donor and osmolyte [3,78,79]. It plays a key role in enhancing energy metabolism, growth performance, and carcass traits. As a methyl donor, betaine contributes to essential biochemical processes such as the remethylation of homocysteine to methionine, thereby sparing methionine and choline for other physiological functions [80,81]. As an osmolyte, it helps maintain cellular water balance under osmotic stress, improving intestinal integrity, nutrient absorption, and overall metabolic resilience [78,82].

Numerous studies have demonstrated that dietary betaine supplementation improves average daily gain (ADG) and feed conversion ratio (FCR), particularly under conditions of heat stress or nutrient restriction [3,78,83,84]. However, responses may vary depending on factors such as diet composition, environmental conditions, and genetic merit of the animals. Notably, there is no evidence that betaine supplementation enhances fat-free mass in humans [22,74]. Betaine has been shown to reduce fat deposition and promote lean tissue accretion by modulating hepatic lipid metabolism and stimulating fatty acid oxidation. Improvements in carcass characteristics—such as reduced backfat thickness and increased lean mass—are particularly evident when betaine is administered during specific growth phases (e.g., between 10 and 16 weeks of age in sows) [79,83,84,85,86,87,88]. Meta-analyses further confirm betaine’s beneficial effects on meat quality, including improved colour, enhanced water-holding capacity, and reduced cooking and drip losses [79]. Improvements have also been reported in blood biochemical parameters, including reductions in plasma urea and cholesterol and increases in albumin and total protein levels—indicators of enhanced protein metabolism [84]. Through its osmolytic function, betaine helps maintain cellular hydration and volume under osmotic stress, particularly in renal and intestinal tissues. This protective effect preserves cellular function by replacing inorganic ions, stabilising proteins and membranes, and reducing energy expenditure associated with ion transport [89,90]. These mechanisms are especially important during gastrointestinal challenges such as coccidiosis, where betaine supplementation has been shown to improve gut morphology, reduce lesion severity, and enhance nutrient absorption [91,92]. Its ability to reduce diarrhoea and strengthen intestinal integrity has been documented in several studies [93,94]. Given the anticipated restrictions on in-feed antibiotics, betaine is increasingly regarded as a promising bioactive compound for promoting gut health [78].

From a practical standpoint, recommendations for betaine use vary across different stages of pig production. Identifying the most critical phases for supplementation is essential for optimizing its functional benefits while reducing the duration of inclusion and, consequently, production costs [95]. Reported effective inclusion rates are approximately 0.1% in diets for piglets and grower pigs, and up to 0.2% in breeder diets. The effectiveness and cost-efficiency of betaine depend largely on the timing and duration of supplementation. The critical phases for betaine supplementation are the first week after weaning for weaner pigs (4–10 weeks), especially for light weight pigs (it is not beneficial when other methyl donors are sufficient), the growth phase for fattening/finisher pigs (10–16 weeks) (it is recommended in female grower pigs for backfat reduction), and in sows during summer lactation and gestation to manage heat stress [96,97].

### 5.2. Poultry

Dietary betaine supplementation has demonstrated beneficial effects across various poultry species (Table 1), improving performance [98,99,100,101,102], nutrient utilization [103,104], and physiological resilience, particularly under environmental stress conditions [105,106,107]. In broiler chickens, betaine enhanced growth performance indicators such as feed intake, body weight gain, and FCR [108,109], including under heat stress [110]. Supplementation has also been associated with increased pectoral muscle yield [106,108,111] and improved performance under disease challenges, including coccidiosis [112]. These effects may be attributed to betaine’s role as an osmolyte, preserving intestinal epithelial integrity, enhancing nutrient digestibility, and promoting the expansion of intestinal mucosa [113]. Furthermore, betaine acts as a methyl donor, potentially improving energy efficiency by conserving ATP normally expended for Na⁺/K⁺ pump function, especially under thermal stress [114,115]. Enhanced availability of sulfur amino acids has also been noted [116]. Betaine’s positive influence extends to gut microbiota modulation. Supplementation increased beneficial microbial populations such as Enterococci, while reducing total bacterial counts in the crop [113]. It also elevated concentrations of short-chain fatty acids (SCFAs), including acetic and propionic acids, essential for beneficial bacteria such as Lactobacillus and Bifidobacterium [105,117]. However, not all studies report consistent benefits; some have shown no significant effects on performance at certain dietary inclusion levels [118,119,120]. In laying hens, betaine has shown promising effects on egg production, FCR, and eggshell quality, particularly under heat stress [102,104]. It improved laying performance, hormonal profiles, and bone strength [113,121]. The compound also mitigated heat stress–induced reductions in vitellogenin and very-low-density lipoprotein levels in egg yolk precursors [113]. When combined with other nutrients (e.g., folic acid, choline, vitamins E and C), betaine further improved performance in heat-challenged layers [104]. These effects appear to be mediated through endocrine modulation, particularly via increased luteinizing hormone, estradiol, melatonin, and follicle-stimulating hormone levels [113].

In ducks, particularly under heat stress conditions, dietary or water-based betaine supplementation has been effective in improving live weight gain (LWG), feed efficiency, and physiological biomarkers [117]. Combination with antioxidants, such as Vitamin C, further enhanced these effects. Betaine also improved laying, hatchability, semen quality, nutrient digestibility, and economic performance in ducks under summer conditions [122]. Interestingly, when compared with DL-methionine, betaine proved more effective in enhancing feed efficiency, carcass traits, and growth in starter ducks [98]. In quails, results are less consistent. Some studies have reported increased egg weight and body weight gain with dietary betaine supplementation, particularly when combined with specific energy and protein levels [123]. However, other studies observed no significant improvements in egg production during early or peak laying periods [100]. Research on turkeys is limited. However, available evidence suggests that oral administration of anhydrous betaine (2.5 g/L in drinking water) over two days led to a favorable response in 96% of infected birds over 70 days of age [113], indicating potential immunomodulatory and stress-alleviating properties worthy of further investigation.

**Table 1:** Effect of betaine supplementation in poultry.

Source	Dosage	Species	Main Effects ^1^	Reference
Feed-grade betaine	0.5 g/kg	Ducks, White Pekin, 1–41 days	BW, BMY	[98]
Water-added betaine	50, 100 g/kg	Broilers, Cobb, 1–42 days	RT, H/Lr	[124]
Feed-grade betaine	1 g/kg	Broilers, Cobb 500, 1–56 days	FCR, BMY	[108]
Feed-grade betaine	0.75, 1.5, 2.25 g/kg	Broilers, Ross 308, 1–42 days	BWG, FCR, Nd	[103]
Feed-grade betaine	0.06, 1.2 g/kg	Broilers, Ross 308, 1–42 days	RtC	[112]
Feed-grade betaine	0.8 g/kg	Broilers, Cobb 400, 1–42 days	BWG, Sbp	[125]
Feed-grade betaine	0.75, 1.5, 2.25 g/kg	Turkey, Bronze, 12–20 weeks	BWG, Sbp, CW, %D	[99]
Feed-grade betaine	1 g/kg	Laying hens, Mandarah, 32–48 weeks	BWG, EP, FI, FCR, Nd	[104]
Feed-grade betaine	0.7, 1, 1.3 g/kg	Ducks, NR, 1–42 days	BWG, FI, FCR, Bh, SCFA	[105]
Feed-grade betaine	1.2 g/kg	Ducks, Cherry Valley, 1–42 days	BW, FI, FE, Bh, SCFA	[117]
Feed-grade betaine	0.6, 12 g/kg	Japanese quails, 42–98 days	Nd, FI, EP, EW, FCR, EQ	[123]
Betaine hydrochloride	0.25 g/kg BW	Broilers, Ross, 1–42 days	Bh, ADG, FCR, FI, WI	[109]
Feed-grade betaine	1 g/kg	Broilers, Ross 308, 1–42 days	ADG, FCR, BMY, RR	[106]
Anhydrous betaine	0.5, 1, 2 g/kg	Broilers, Huaixiang, 1–28 days	BWG, FI, IH	[126]
Anhydrous betaine	1.2 g/kg	Laying quails, 23–27 weeks	Nd, FI, EP, EW, FCR, EQ	[100]
Feed-grade betaine	0.5, 1, 2 g/kg	Broilers, Huaixiang, 1–35 days	BWG, FI, NR	[127]
Feed-grade betaine	0.8 g/kg	Ducks, NR, 1–42 days	BW, FI, FCR, Bh, Im, SCFA	[122]
Anhydrous betaine	1, 2, 3 g/kg	Broilers, Ross 308, 1–35 days	M, BMY, BMfl, BMpH, a*, Dl, CL, SF, OS	[111]
Feed-grade betaine	1 g/kg	Broilers, Huaixiang, 5–15 weeks	IBF	[107]
Active natural betaine	1 g/kg	Broilers, Ross 308, 1–35 days	BWG, FI, FCR, IH	[101]
Water-added betaine	1 g/L	Broilers, Ross 308, 21–35 days	%D, CC, WHC, CL, Mt	[128]
Feed-grade betaine	2 g/kg	Broilers, Ross 308, 1–35 days	BWG, FI, Sbp, Mmp, Ir	[110]
Feed-grade betaine	8 g/kg	Laying hens, Hy-Line Brown, 21–27 weeks	Lr, EQ	[102]
Feed-grade betaine	1.5, 3 g/kg	Broilers, NR, 1–21 days	FCR, Sbp, Lbp	[129]
Feed-grade betaine	0.5, 1, 1.5 g/kg	Broilers, Caribro-vishal, 0–42 days	BWG, FI, FCR, CMIR, Gm; Bh, BMW	[130]
Feed-grade betaine	1, 3 g/kg	Laying hens, Hy-Line Brown, 71–77 weeks	Im, Lr; Mc	[121]

^1^ %D: dressing percentage; a*: meat redness; ADG: average daily weight gain; Bh: blood hematology; BMfl: Breast muscle fibre length; BMpH: fresh breast muscle pH; BMW: breast muscle weight; BMY: breast muscle yield; BWG: body weight gain; CL: cooking loss; CMIR: cell mediated immune response; CW: carcass weight; Dl: drip loss; EP: Egg production; EQ: egg quality; EW: egg weight; FI: Feed intake; Gm: Gut morphology; H/Lr: Heterophil/lymphocyte ratio; IBF: intestinal barrier function; IH: Intestinal histomorphology; Im: Intestinal morphology; Ir: Immune response; Lbp: Liver biochemistry parameters; Lr: Laying rate; M: Mortality; Mc: Microbiota composition; Mmp: muscle mineral profile; Mt: meat texture; Nd: Nutrient digestibility; NR: Nutrient Retention; OS: oxidative status; RR: respiration rate; RT: rectal temperature; RtC: Resistance to coccidiosis infection; Sbp: Serum biochemical parameters; SCFA: Short Chain Fatty Acid Profile of Ceca; SF: Shear force; WHC: water-holding capacity; WI: water intake.

### 5.3. Rabbits

Heat stress leads to major physiological and reproductive issues in rabbits: they are particularly vulnerable due to their poor sweating capacity and difficulty in dissipating heat. A temperature-humidity index (THI) ≥ 30 is classified as severe heat stress, resulting in reduced performance and health problems [131]. As regards the use of betaine in rabbit diet formulation, in recent decades, numerous experiments have been performed on rabbits, in particular under heat stress. The major goal, with a feed inclusion rate that varied from 0.5 to 2 g/kg/day, was to evaluate the positive effects of dietary betaine alone, or in association with other ingredients, to enhance resistance to heat stress by improving rabbit performance, nutrient digestibility, intestinal health, immune response, and antioxidant status [131,132,133,134,135,136,137,138]. As already mentioned in the previous sections, the criticism highlighted in these studies is precisely that of the use of betaine under specific stressed environmental conditions and not under normal conditions.

### 5.4. Horses

While nutritional supplements and specific feeding strategies are widely used in equine sports, few have demonstrated clear, reliable performance benefits in horses. In the past, some research groups [139,140] have investigated the use of betaine, as an antioxidant, in cryopreservation of stallion semen, with controversial results. The use of betaine supplementation alone, or in association with other metil donors, in trained horses has been studied with the aim of enhancing performance; while some earlier studies [141,142,143] showed no clear effect of DMG (Dimethylglycine) on equine performance, the study of de Oliveira et al. [144] supports the hypothesis that longer supplementation periods (20–30 days) may improve lactate metabolism and extend exercise duration, possibly through enhanced activation of the pyruvate dehydrogenase enzyme, aiding glucose oxidation.

### 5.5. Dogs and Cats

The potential benefits of betaine in companion animal nutrition arise from its involvement in three key metabolic domains: (i) its central role in one-carbon metabolism, where it donates methyl groups for essential biochemical processes; (ii) its influence on fatty acid metabolism, affecting lipid utilization and distribution; and (iii) its function as an osmolyte, supporting cellular homeostasis under osmotic stress [145]. These functions are particularly relevant in the cat, given its unique metabolic profile as an obligate carnivore with elevated protein requirements and limited flexibility in amino acid metabolism [146].

As a methyl donor, betaine supports the remethylation of homocysteine to methionine, a pathway critical for sustaining methylation-dependent processes such as DNA synthesis, gene expression, and antioxidant defence. This may also help spare dietary methionine, an essential and often limiting amino acid in feline diets. Furthermore, cats display distinct features within their one-carbon metabolism that may increase their reliance on dietary methyl donors. The synthesis of felininine, a sulfur-containing amino acid used in urinary pheromone signalling, along with a limited capacity for taurine biosynthesis and elevated methionine adenosyltransferase (MAT) activity, all contribute to an increased demand for methylation [146].

In most studied species, betaine supplementation is known to support lipid metabolism by enhancing fatty acid uptake and oxidation and improving intramuscular fat composition. However, in cats, dietary polyunsaturated fatty acid (PUFA) intake is associated with a reduction in circulating betaine, suggesting a distinct metabolic demand for methyl donors during lipid metabolism and highlighting species-specific differences in fatty acid handling [147].

Recent studies have highlighted the interactions between dietary betaine and PUFAs in modulating lipid and one-carbon metabolism in dogs and cats [148,149]. In dogs, 60 days of supplementation with betaine (ranging from 0.46% to 0.58% diet as fed), alone or in combination with flaxseed (a source of alpha-linolenic acid, ALA, 0.83% diet) or fish oil (providing eicosapentaenoic acid [EPA, 0.15% diet] and docosahexaenoic acid [DHA, 0.10% diet]), significantly altered circulating metabolites. Betaine increased plasma concentrations of key one-carbon metabolites including dimethylglycine, methionine, and N-methylalanine, while also enhancing EPA synthesis from ALA and reducing circulating carnitine and acylcarnitines, suggesting a shift toward enhanced fatty acid utilization and mitochondrial efficiency [148].

In cats, similar dietary interventions revealed species-specific metabolic responses. Betaine supplementation for 60 days (0.62% diet as fed), particularly in conjunction with fish oil (EPA 0.15%, DHA 0.11%), elevated plasma levels of betaine, dimethylglycine, and several long-chain fatty acids, including DHA and ALA [149]. These findings are supported by an earlier study, which demonstrated that dietary enrichment with either fish oil (as a source of EPA/DHA) or arachidonic acid in cats resulted in significant reductions in circulating betaine, dimethylglycine, and sarcosine, suggesting increased utilization of one-carbon donors during PUFA metabolism [147]. These results highlight a potential nutritional demand for enhanced one-carbon metabolism support, such as betaine, when high levels of PUFA are included in feline diets.

Interestingly, the effect of betaine on circulating fatty acid profiles appears to diverge between dogs and cats. In dogs, the combination of betaine and ALA promoted an intermediate increase in EPA levels, even without direct fish oil supplementation, pointing to a possible role for betaine in enhancing fatty acid desaturation and elongation pathways [148]. Conversely, in cats, no such increase in EPA was observed with added ALA, even in the presence of betaine [149]. While betaine influenced circulating PUFA concentrations in both species, the metabolic response to ALA was more pronounced in dogs. These findings reflect underlying species differences, likely associated with the cat’s limited delta-6 desaturase activity and reduced capacity to convert precursor fatty acids into longer-chain forms. Furthermore, reductions in acylcarnitines and dicarboxylates suggested improved fatty acid oxidation and mitochondrial function [149].

As an osmolyte, betaine plays a crucial role in maintaining cellular homeostasis during osmotic stress. This is particularly important for kidney health and function, as betaine helps mitigate the damage caused by hypertonicity and the accumulation of homocysteine. Research has shown that betaine can reduce hypertonicity-induced cell death in renal cell lines derived from dogs, offering significant insights into its potential for managing kidney disease [150].

Recent studies investigated the effects of diets enriched with betaine (0.5% of diet) and different soluble fibre supplementations (short-chain fructooligosaccharides, apple pomace, and oat beta-glucan) on dogs and cats with spontaneous chronic kidney disease (CKD) in different stages [151,152]. In CKD dogs, the dietary treatment with betaine and increased soluble fibre was shown to decrease creatinine levels and increase circulating methionine [151]. In the one-carbon metabolism pathway, betaine donates a methyl group to convert the uremic toxin homocysteine into methionine, during which it is transformed into dimethylglycine. This metabolite was shown to increase in CKD cats when fed a diet enriched with betaine and apple pomace [152]. These findings suggest the potential of dietary interventions with betaine, offering a promising approach to managing renal disease in dogs and cats.

Collectively, these findings highlight betaine’s capacity to modulate both one-carbon and lipid metabolism in dogs and cats.

### 5.6. Fishes

Betaine plays a crucial role in aquaculture, bringing a variety of positive effects, among others, by enhancing feed intake, growth performance, immunity, osmoregulation, and metabolic functions [153,154,155,156,157,158,159] (Table 2). Betaine is typically provided to aquatic species through feed supplementation, ensuring uniform distribution of the nutrient or feeding stimulant to all animals during feeding. However, the study by Lu et al. [160] showed how intraperitoneal betaine injection can also be successfully used as a method to provide betaine to ayu (*Plecoglossus altivelis*). Despite this, the industry-standard method for betaine delivery remains direct feed supplementation in commercial aquafeed.

The primary benefit of betaine in aquaculture is its role as a feed attractant, improving the palatability and acceptance of aquafeeds, particularly when alternative plant-based protein sources are used [161]. Betaine stimulates the cephalic reflex, enhancing feed intake and nutrient absorption. Studies have shown that *Salmo trutta* fed with 1.4% betaine in plant-based diets exhibited improved feed acceptance, growth performance, immune response, antioxidant defense, and digestive activity [156,161]. Similarly, *Oncorhynchus mykiss* demonstrated increased feed intake and utilization when supplemented with 1.5% betaine [162]. The positive effects of dietary betaine supplementation on growth have been observed in various other species, including Nile trout *Oreochromis niloticus* [158,163], common carp *Cyprinus carpio* fingerlings [164], rainbow trout *Oncorhynchus mykiss* [162,165,166], and *Lithobates catesbeianus* [167]. Furthermore, betaine has also been used for Sea Bream larvae (*Sparus aurata*) as a potent chemical feeding stimulant, significantly enhancing microdiet ingestion rates when included in the rearing environment, either alone or synergistically with other attractants like arginine, alanine, and glycine [168,169].

Beyond its role as a feed attractant, betaine functions as an osmoprotectant, helping aquatic species maintain cellular balance under stressful environmental conditions, such as fluctuating salinity levels. Research has demonstrated that dietary supplementation with betaine improves survival rates when stressed by water salinity variation. For instance, *Labeo bata* fed with 0.025% betaine exhibited enhanced survival in brackish water conditions [170], while *Oreochromis aureus* showed increased survival when reared in seawater with 1.0% betaine supplementation [171].

In addition to its role in osmoregulation, betaine significantly influences metabolic functions, particularly in protein and lipid metabolism. As a methyl donor, betaine modulates fat accumulation and lipid metabolism, reducing hepatic fat deposition. Studies have shown that betaine supplementation downregulates the expression of fatty acid synthase (FASS) and acetyl-CoA carboxylase (ACC) genes, mitigating hepatic steatosis in species like *Carassius auratus gibelio* [172]. It also promotes fatty acid oxidation by upregulating carnitine palmitoyltransferase 1 (CPT1) and peroxisome proliferator-activated receptor alpha (PPARα) genes. In black seabream *Acanthopagrus schlegelii*, betaine supplementation in high-fat diets increased Sirt1 expression, a gene responsible for enhancing lipid breakdown and reducing fat accumulation in the liver [173].

Betaine also plays a critical role in enhancing immune function and resistance to disease. It has been shown to boost antioxidant enzyme activity, such as superoxide dismutase (SOD) and catalase (CAT), thereby protecting cells from oxidative stress in Caspian trout *Salmo trutta* [161]. Research on Ayu *Plecoglossus altivelis* demonstrated that betaine injection stimulated the immune response, enhancing monocyte/macrophage activity against *Vibrio anguillarum* [160]. Similarly, in *Macrobrachium rosenbergii*, dietary supplementation with 0.5% betaine significantly improved innate immunity against *Vibrio cholera*, leading to better survival rates [157]. Betaine also improves the phagocytic activity and bacterial-killing ability of immune cells, providing an additional layer of defense against infections in Nile tilapia (*Oreochromis niloticus*) [174].

Another significant benefit of betaine supplementation in aquaculture is its impact on meat quality. By influencing muscle composition, betaine increases the ratio of beneficial fatty acids while reducing saturated fat content in blunt snout bream fed a high-fat diet [154,157]. This not only improves the nutritional value of aquaculture products but also enhances their commercial appeal.

Overall, betaine is a multifunctional feed additive in aquaculture, contributing to improved growth, feed utilization, immune function, osmoregulation, metabolism, and meat quality. The optimal dietary levels of betaine, typically ranging between 0.5% and 1.5%, provide the best results, while excessive supplementation may negatively affect survival rates. Its versatility makes it a valuable component in aquafeeds, helping to optimize production efficiency and the overall health of farmed aquatic species. In light of the increasing environmental stressors driven by climate change—such as rising water temperatures, fluctuating salinity levels, ocean acidification, and increased pathogen pressure—future research on betaine in aquaculture should focus on its potential as a strategic additive for enhancing resilience in farmed aquatic species. Given its proven roles in osmoregulation, immune modulation, and stress mitigation, betaine represents a promising tool in developing adaptive feeding strategies to support fish health and performance under climate-induced challenges. Notably, the Mediterranean Sea, home to economically important species such as European sea bass (*Dicentrarchus labrax*) and gilthead sea bream (*Sparus aurata*), is considered a climate change hotspot, where marine ecosystems are already experiencing rapid shifts in temperature and salinity. Despite this, studies examining the effects of betaine supplementation in Mediterranean aquaculture species remain scarce. This represents a critical knowledge gap, especially given the region’s vulnerability and its reliance on aquaculture as a major food and economic resource. Future investigations should not only evaluate species-specific responses to betaine under stress conditions but also explore the molecular mechanisms involved in its protective effects. Understanding how betaine interacts with physiological stress responses in Mediterranean species could significantly contribute to the development of climate-resilient aquaculture practices in this increasingly fragile marine environment.

**Table 2:** Overview of the recommended doses of betaine in different water animals and observed positive effects.

Species	Betaine Recommended	Observed Effects	Source
Hybrid Grouper *Epinephelus lanceolatus ♂ × Epinephelus fuscoguttatus ♀*	0.5%	Improved feed efficiency, protein and methionine retention, growth performance (SGR), and reduced ammonia excretion.	[159,175]
Rainbow Trout (*Onchorincus mykiss*)	0.5–1%	In combination with 25% soybean meal, positively influenced growth, feed utilization, and fatty acid profiles.	[156,166,176]
Nile Tilapia (*Oreochromis niloticus*)	0.05%	Improved weight gain, body protein content, reduced FCR and body fat.	[158,163]
Common Carp (*Cyprinus carpio*)	0.3%	Growth performance (BWG, DWG, FCR, FCE, RGR%, SGR%) improved and enhanced digestive enzyme activity.	[177,178,179]
Caspian Trout (*Salmo trutta*)	1.4%	Higher weight gain, SGR, and PER compared to the baseline (plant-protein diet). Digestive enzyme activity (lipase, amylase) increased.	[161]
Giant Freshwater Prawn (*Macrobrachium rosenbergii*)	0.5%	Improved weight gain, FCR, daily growth, and feed intake vs. control. Digestive enzyme activity was higher. Survival rate under bacterial stress also increased.	[157]
Black Tiger Prawn (*Penaeus monodon*)	2%	Effects on intestinal histology, lipid metabolism, and immune response.	[180,181]
Pikeperch (*Sander luciperca*)	2%	Increased palatability and acceptability of food for fingerlings.	[182,183]
Ayu (*Plecoglossus altivelis*)	100–200 mg/kg (injection)	Protection against *Vibrio anguillarum.*	[160]
Bullfrog Bass *(Lithobates catesbeianus)*	0.4%	Increased whole-body protein deposition.	[167]

## 6. Betaine in Ruminant Nutrition

Betaine, has gained increasing attention in ruminant nutrition due to its multifaceted roles in enhancing metabolic efficiency, maintaining osmotic balance, and supporting overall animal performance. Naturally derived from sugar beet processing or synthesized endogenously from choline oxidation, betaine is widely used as a feed additive in the form of anhydrous betaine, betaine monophosphate, or betaine hydrochloride [184]. It functions primarily through three key mechanisms: acting as an osmolyte, serving as a methyl donor, and functioning as a molecular chaperone [185,186]. Additionally, its role as a methyl donor in one-carbon metabolism supports critical physiological functions, including growth, lactation, and liver function, by donating methyl groups to methionine via S-adenosylmethionine [187]. These metabolic contributions allow betaine to spare choline for other essential processes, enhancing overall metabolic efficiency [78]. Due to these properties, betaine supplementation has been investigated for its potential to improve milk yield and composition, growth performance, and carcass quality in ruminants. Its benefits are particularly evident under both thermoneutral and heat stress conditions, where it aids in reducing energy expenditure, protecting gut tissues, and maintaining osmotic balance [188]. This section will provide an overview of betaine’s physiological roles in ruminants, its dietary sources, and the latest findings on its impact across different ruminant species, with a focus on dairy and beef cattle, goats, and sheep.

### 6.1. Betaine and Rumen Microorganisms

Betaine can influence the growth and activity of the microbes in the gastrointestinal tract of cattle due to its ability to maintain native protein structure and prevent cell lysis. Under stress conditions, microbial cells increase the uptake of exogenous betaine, which reduces its availability to the rumen epithelium. However, some betaine can escape the rumen and reach the duodenum [189]. Betaine acts as a promoter of bacterial growth under osmotic stress conditions and can be metabolized and converted to acetate in the rumen, contributing to milk fat synthesis [190], which suggested an interaction between betaine and fatty acid production in dairy cows.

Mahmood et al. [191] demonstrated that betaine has numerous applications in in vitro rumen fermentation studies. It stabilizes the bacterial community and enhances the ruminal fermentation process. Their study explains that betaine, due to its chemical structure with methyl groups, can act as a methyl donor for methanogenesis and directly affect methylotrophic archaea. Archaea are more stress-tolerant microorganisms than bacteria, but they are sensitive to several external factors, including dietary components. These factors lead to cellular dehydration, cell death, dysbiosis, and reduction of animal production and health. Archaea represent only 3% of microbial biomass in the rumen, and they occupy an important ecological position in the rumen microbiome and rumen fermentation [191,192]. A group called “rumen cluster C” Thermoplasmata has been discovered as a new order of methanogens. They were classified as methylotrophic methanogens and occupied a different niche separate from the other methanogens, as suggested by Poulsen et al. [193]. Thermoplasmata can obtain energy and carbon from methylamine derived from the degradation of betaine and choline. The use of methylamine by Thermoplasmata impacts the metabolic process of rumen bacteria producing ammonia nitrogen [193]. This production leads to a positive change in the protease activity of rumen bacteria such as Butyrivibrio fibrisolvens, indicating a stimulatory effect on crude protein degradation [194]. The ammonia nitrogen produced does not alter its concentration in the rumen, suggesting that it could be used for microbial protein synthesis. Betaine also influences the activities of protozoa and fibrolytic bacteria such as Ruminococcus albus and Fibrobacter succinogenes, increasing fiber digestibility [195].

### 6.2. Betaine Supplementation in Cattle

In cattle, betaine supplementation has been explored mainly in dairy and beef production systems. Studies have investigated its impact on DMI, milk yield, and milk composition in dairy cows, particularly under thermoneutral and heat-stress conditions [185,196]. Betaine functions as an osmoprotectant, helping maintain cellular hydration and reducing energy expenditure under environmental stressors [78]. Additionally, it acts as a methyl donor in one-carbon metabolism, potentially sparing methionine for protein synthesis and improving lactation performance [195]. While several studies have demonstrated a positive impact of betaine on milk yield and fat content, findings remain inconsistent, with some reports indicating no significant effects on performance parameters [197,198]. Furthermore, research has highlighted the importance of betaine availability in the rumen and its influence on microbial fermentation, suggesting the need for targeted formulations to enhance its efficacy [199]. A summary of key studies on betaine supplementation in cattle is provided in Table 3.

Betaine supplementation has been shown to enhance DMI in both heat-stressed and non-heat-stressed dairy cows. This finding is consistent with previous studies in which dietary betaine was provided [185,209]. Notably, no significant differences were observed in the effects of betaine on DMI between heat-stressed and non-heat-stressed cows. The precise mechanism by which betaine improves DMI remains unclear; however, it is hypothesized that betaine may influence ruminal fermentation by serving as a source of either rumen-available nitrogen [204] or methyl groups [212]. The degradation of betaine by ruminal microbes has been previously reported, supporting its potential role in modifying ruminal fermentation dynamics [213]. Another proposed mechanism is the enhancement of ruminal nitrogen availability, which could promote the proliferation of beneficial microbes and, consequently, improve DMI and the total tract digestibility of NDF and ADF [204]. Betaine supplementation in heat-stressed dairy cows has been associated with improved total tract digestibility of dry matter (DM), organic matter (OM), crude protein (CP), NDF, and ADF. These findings are in agreement with those of Wang et al. [204], who reported similar improvements in nutrient digestibility upon betaine inclusion in the diet. The beneficial effects of betaine may be attributed to its dual role as an osmolyte and methyl donor, which helps mitigate heat stress, maintain optimal rumen pH, and enhance the microbial composition in the rumen [78]. Several studies [204,206,210] have demonstrated that betaine supplementation during the periparturient period and early lactation increases milk yield in dairy cows, regardless of ambient temperature conditions. A recent meta-analysis by Abhijith et al. [186] further corroborated this effect, reporting an average increase of 1 kg of milk per day in betaine-supplemented cows compared to controls. This positive impact on milk yield has also been observed in cows under heat stress conditions [189,196] and in grazing cows during the summer. Notably, the increase in milk production is evident in both heat-stressed cows (+1.67 kg/day) and cows housed under thermoneutral conditions (+1.03 kg/day) [198]. Additionally, energy-corrected milk (ECM) and fat-corrected milk (FCM) production were enhanced in betaine-supplemented cows [190,196,207]. This effect is likely due to betaine’s role in the remethylation of homocysteine to Met, sparing Met from acting as a methyl donor. Wang et al. [210] suggested that betaine donates a methyl group to facilitate the conversion of homocysteine to Met, particularly in cases of dietary Met insufficiency. However, the effects of betaine supplementation on milk production may vary depending on dietary composition and lactation stage. For example, Davidson et al. [203] found no significant changes in milk yield or milk solids when betaine was supplemented at 45 g/day during early lactation, possibly because the control diet was not deficient in Met. Such discrepancies highlight the importance of dietary composition in determining betaine’s efficacy. Betaine supplementation has also been associated with improvements in milk quality, particularly an increase in milk fat content [189,190,196,204,207]. This enhancement may be linked to betaine’s ability to stimulate ruminal acetate production [189,206] and improve the digestibility of OM, CP, NDF, and ADF [209]. Nevertheless, some studies have reported no significant effects of betaine on milk fat percentage (e.g., Peterson et al., [197], with 100 g/day of rumen-unprotected betaine). Such inconsistencies may be attributed to differences in basal diet composition rather than lactation stage per se. Moreover, Wang et al. [210] observed that supplementing 20 g/day of rumen-protected betaine reduced milk somatic cell count (SCC), suggesting a potential role in maintaining mammary gland health. Although the precise mechanisms remain unclear, possible explanations include betaine’s ability to support mammary cell proliferation and maintain cellular osmolarity [207]. Additionally, studies have suggested that betaine supplementation can reduce oxidative stress in mammary epithelial cells under heat stress conditions [214] and decrease pro-inflammatory cytokine production, indicating its potential as a feed additive for mastitis management [215]. Another potential mechanism underlying betaine’s beneficial effects on nutrient digestibility and animal performance is its influence on mitochondrial function. Betaine has been shown to enhance mitochondrial fission and fusion dynamics, thereby promoting cell homeostasis and survival [216]. Furthermore, betaine has been reported to stabilize cellular and subcellular membranes by restoring both enzymatic and non-enzymatic antioxidants, positively regulating mitochondrial function [217,218]. Given that part of betaine can be metabolized by rumen microbes [219], it is plausible that betaine improves rumen microbial function, thereby contributing to enhanced feed efficiency and overall performance. Beyond its effects on nutrient digestibility and milk production, betaine supplementation influences plasma metabolic parameters. Wang et al. [210] reported a significant increase in plasma glucose concentrations following supplementation with 20 g/day of rumen-protected betaine. Similarly, Hall et al. [189] observed elevated plasma glucose and insulin levels in heat-stressed cows supplemented with betaine. The increased plasma glucose concentration may result from a reduction in peripheral glucose uptake [210]. Additionally, several studies have reported a decrease in non-esterified fatty acids (NEFA) and plasma triglyceride concentrations in betaine-supplemented cows compared to controls [208,210]. Given that NEFA levels reflect energy metabolism balance, their reduction suggests improved dietary energy utilization. Wang et al. [204] observed a linear decrease in plasma NEFA concentrations with increasing levels of betaine supplementation (50, 100, and 150 g/cow/day), potentially due to increased NEFA uptake by the mammary gland for milk fat synthesis, as supported by reports of increased milk fat content [185,207]. Furthermore, the observed reduction in plasma triglycerides [210] may be attributed to their transport to the liver for metabolism into NEFA, facilitated by enhanced fatty acid absorption and methyl group availability for chylomicron synthesis and secretion.

### 6.3. Betaine Supplementation in Small Ruminants

The effects of betaine supplementation in small ruminants, including sheep and goats, have been examined primarily in the context of heat stress mitigation and carcass quality improvement. As an osmolyte, betaine contributes to thermoregulation by reducing endogenous heat production and preserving cellular integrity. Studies in sheep and goats suggest that betaine supplementation can enhance nutrient utilization efficiency, improve growth performance, and support lactation under challenging environmental conditions. However, similar to cattle, responses to betaine supplementation in small ruminants appear to be dose-dependent and variable across different production systems. Research gaps remain in understanding its long-term effects on metabolic pathways and optimal dietary inclusion levels. Table 4 presents a compilation of studies examining betaine supplementation in small ruminants.

Several authors [219,220,221] have reported an increase in milk yield and milk solids, consistent with findings in dairy cows. Specifically, Fernández et al. [219] observed an increase of 0.37 kg/day in milk production in goats fed a betaine-supplemented diet. Supporting this result, Fernández et al. [220] reported a comparable increase in milk yield (0.37 kg/day) after three months of betaine supplementation at a rate of 2 g/kg of diet. Regarding milk quality, the literature presents conflicting data. Ghoneem and El-Tanany [221] observed a 10% increase in milk production when using dehydrated condensed molasses fermentation solubles and a 7% increase with anhydrous betaine. These authors also reported an increase in fat-corrected milk (FCM) yield (1.46 vs. 1.29 for betaine supplementation and control, respectively). This increase aligns with the observed rise in milk fat percentage (4.47% and 4.55% vs. 4.26% in the control) and total solids, the latter of which was statistically significant. These findings are consistent with an increase in ruminal acetate concentration (28.11 and 29.05 mmol/L, respectively) compared to the control group (25.96 mmol/L). It has been suggested that betaine supplementation can alter rumen fermentation patterns, favoring a higher acetate concentration, which may in turn promote fat synthesis in the mammary gland [209]. Similarly, Fernández et al. [220] observed an increase in milk fat percentage (4.69% vs. 4.40% for betaine vs. control, respectively) in lactating Murciano-Granadina goats fed betaine-supplemented diets. However, others [219,222] found no significant differences in the chemical composition of milk. An interesting aspect of milk composition is its fatty acid profile. Fernández et al. [220] reported significantly higher (*p* < 0.05) levels of caprylic (C8:0) and capric (C10:0) fatty acids in the betaine-supplemented group compared to the control (3.03% vs. 2.8% for caprylic acid; 11.14% vs. 10.05% for capric acid). Additionally, betaine supplementation significantly increased (*p* < 0.05) the concentration of short-chain fatty acids (mainly C8:0, C10:0, C10:1, and C12:0), as well as C18:3. Conversely, the levels of other C18 fatty acids (except for C18:3) decreased, with a significant reduction observed for C18:1 (*p* < 0.05). Several studies on betaine supplementation in small ruminants have focused on growth performance and birth outcomes [223,224,225,226]. For instance, Sahraei et al. [227] reported that betaine supplementation in pregnant ewes tended to increase lamb birth weight (4.41 vs. 3.95 kg). Additionally, lamb body weight and average daily gain (ADG) at 56 days of age were significantly different between groups (ADG: 221 ± 18 g in the control group vs. 224 ± 23 g in the betaine group). Similar findings were reported by Brougham et al. [226], who observed that lambs born to betaine-supplemented ewes tended to have higher body weights at weaning. In another study, Brougham et al. [226] investigated whether maternal betaine supplementation during pregnancy could improve twin lamb body weight, thermoregulatory capacity, time to stand and suckle, colostrum intake, and survival from birth to weaning. Supplementing ewes with 4 g/day of betaine during the second half of pregnancy increased twin lamb survival up to day 7 and reduced the time required to initiate suckling. However, feeding ewes 2 g/day of betaine throughout pregnancy improved postnatal growth at weaning but reduced twin lamb survival rates and increased the time needed to reach key behavioral milestones. Overall, betaine supplementation during late gestation appears to have a positive effect on early postnatal twin lamb survival.

Regarding the effect of betaine supplementation in lambs and meat goats [219,223,224,225,228,229], a review of the literature does not show a clear effect on weight gain and final body weight following betaine supplementation, except for Dong et al. [225], who reported an improvement in lamb growth performance. These authors also observed a positive trend in average daily feed intake. Although growth performance was not consistently enhanced, several studies have shown improvements in meat quality, particularly a reduction in subcutaneous fat and intramuscular lipids, with limited effects on fatty acid composition [223]. Banskalieva et al. [224] reported that 3 g/day of rumen-protected betaine supplementation could improve the growth of goats, possibly by increasing peripheral tissue lipolysis or decreasing lipogenesis. Conversely, Fernández et al. [219,223] found that 2 g/kg dietary rumen-protected betaine supplementation had no effect on growth performance and carcass fatness in both male and female lambs. However, betaine supplementation (1.1 g/day unprotected betaine or 1.1, 2.2, or 3.3 g/day rumen-protected betaine) increased ADG and ADFI in lambs. The observed discrepancies among studies may be due to differences in experimental subjects and feeding doses. There is considerable interest in the effects of betaine on lipid metabolism in muscles. Some studies suggest that betaine accelerates fatty acid oxidation and promotes their transfer to meat, leading to a redistribution of body fat [223]. In contrast, other studies indicate that betaine has a limited effect or may reduce intermuscular fat content [219]. However, few studies have explored betaine’s impact on the fatty acid composition of lamb meat. Liu et al. [230] found that 8 g/day of betaine supplementation had no effect on fatty acid content in the longissimus muscle of finishing sheep. However, Dong et al. [225] reported that betaine supplementation increased polyunsaturated fatty acids (PUFA) in the gluteus muscle. Additionally, rumen-protected betaine supplementation decreased the saturated fatty acid (SFA) content in the longissimus dorsi while increasing unsaturated fatty acids (UFA), monounsaturated fatty acids (MUFA), and the UFA/SFA ratio compared to unprotected betaine treatment.

**Table 4:** Effects of betaine supplementation in sheep and goats.

Source	Dosage	Breed and Category	Diet Type	Main Effects ^1^	Reference
Feed-grade betaine	2 g/kg BW	Manchego, growing lambs	Concentrate mixture and barley straw	FT, NL	[223]
Rumen escape betaine	2 g/kg BW	Manchego, growing lambs	Concentrate mixture and barley straw	ADG, FCR, FT, LMA, CW	[219]
Feed-grade betaine	4 g/kg BW	Murciano–Granadina, lactating goats	Basal pelleted diet and alfalfa hay	MY, MFA,	[220]
Rumen-protected betaine	3 g/d	Boer × Spanish, meat goats	Concentrate mixture and pasture	PTG, NEFA	[224]
Glycine betaine	0.2 g/kg BW	Barbari, non-pregnant goats	Not reported	Heat stress amelioration	[231]
Feed-grade betaine	2, 4 g/d	Merino, ewes	Oat chaff and alfalfa hay	WI, ADG,	[232]
Rumen escape betaine	2, 4 g/d	Merino, pregnant ewes	Basal pelleted diet and oaten hay	LWW, LADG, LBL, BUN, LSur	[233]
Rumen-protected betaine	1.1, 2.2, 3.3 g/d	Hu, growing lambs	Concentrate mixture	FI, ADG, MFA,	[225]
Betaine hydrochloride	5 g/d	Sanjab, pregnant ewes	Concentrate mixture and forages	BHB, MDA,	[227]
Rumen-protected betaine	2, 4, 6 g/d	Hu, growing lambs	Concentrate mixture	Ng, ADG, F/G, GSH-Px, MDA, SOD, CAT,	[228]
Betaine hydrochloride	1, 3 g/d	Hu, growing lambs	Hay-based TMR ^2^	HDL-C,	[229]
Feed-grade betaine	2, 4 g/d	Merino, ewes	Oat chaff and alfalfa hay	I, NEFA,	[234]
Feed-grade betaine	4 g/kg DM	Damascus, lactating goats	Concentrate mixture and Egyptian clover hays	MY, FCM, Nd, NH_3_-N, TrVFA,	[221]
Feed-grade betaine	2, 4 g/d	Merino, ewes and neonatal lambs	Basal pelleted diet and oaten hay	LWW	[235]
Feed-grade betaine	4 g/d	Merino, ewes and neonatal lambs	Pasture and barley grains	RT, LBL; BCS, GL	[226]

^1^ ADG: average daily body weight gain; BCS: Body condition score; BHB: b-hydroxybutyrate; BUN: blood urea nitrogen; CAT: catalase activity; CW: carcass weight; F/G: feed to gain ratio; FCR: Feed conversion rate; FI: Feed intake; FT: Fat thickness; GL: gestation length; GSH-Px: glutathione peroxidase; HDL-C: high-density lipoprotein cholesterol; I: Insulin; LADG: Lamb average daily gain; LBL: Lamb behavioural latencies; LMA: *longissimus* muscle area; LSur: Lamb cumulative survival; LWW: Lamb weight at weaning; MDA: malondialdehyde; MFA: meat fatty acid composition; MY: Milk yield; Nd: nutrient digestibility; NEFA: non-esterified fatty acids; Ng: Net gain; NH_3_-N: ammonia; NL: neutral lipids; PTG: Plasma triacylglycerols; RT: Rectal temperature; SOD: superoxide dismutase activity; TrVFA: Total ruminal volatile fatty acid; WI: Water intake. ^2^ TMR: Total mixed ration.

## 7. Conclusions and Future Perspectives

Betaine has emerged as a biologically active compound with wide-ranging applications in both human and animal nutrition. As a naturally occurring methyl donor and organic osmolyte, betaine exerts crucial physiological functions that support cellular protection, metabolic homeostasis, and organ function. Its ability to mitigate oxidative damage, regulate one-carbon metabolism, and maintain cellular hydration has positioned it as a valuable nutritional supplement in various contexts, particularly under stress-related conditions. In human health, the therapeutic potential of betaine has been demonstrated in a range of disorders, including metabolic-associated fatty liver disease (MAFLD), hyperhomocysteinemia, cardiovascular dysfunction, and renal impairment. The evidence also points to its role in improving body composition, performance, and thermoregulation, although further studies are warranted to clarify optimal dosages and long-term outcomes. Importantly, the interactions of betaine with other nutrients, such as choline, folate, and B-vitamins, underline the need for integrated nutritional strategies rather than isolated supplementation. In the field of animal science, betaine has gained attention as a sustainable and functional feed additive capable of enhancing productive performance and welfare across multiple species. In monogastrics, betaine contributes to improved feed efficiency, growth rate, meat quality, and gastrointestinal integrity—especially under heat stress or pathogenic challenge. In ruminants, it enhances ruminal fermentation, milk yield, nutrient digestibility, and energy balance, particularly in thermally stressed animals. Betaine’s role in sparing methionine and choline in one-carbon metabolism also has implications for feed formulation and cost efficiency. In aquaculture, betaine not only improves feed palatability and growth but also plays a role in osmoregulation, immunity, and stress resilience—traits of increasing relevance under changing climate and water conditions.

Nevertheless, despite the promising results across species and physiological stages, the scientific community must address several unresolved questions. First, a more precise characterization of betaine’s dose-dependent effects is needed, considering factors such as animal age, sex, health status, environmental temperature, and basal diet composition. Second, the variability in response to supplementation observed among studies suggests complex metabolic and microbiota-mediated mechanisms that require deeper molecular understanding. Integration of omics technologies (metabolomics, transcriptomics, proteomics, and epigenomics) may provide critical insights into tissue-specific effects and gene–nutrient interactions modulated by betaine. In addition, the regulatory pathways through which betaine modulates cellular stress responses, mitochondrial bioenergetics, and epigenetic modifications remain partially unexplored. These represent fertile grounds for future investigation, particularly in the context of chronic inflammation, metabolic dysregulation, and reproductive efficiency. Moreover, there is increasing interest in how betaine may interact with the gut–organ axis, influencing systemic health via microbiota-mediated pathways. From a sustainability perspective, the use of betaine derived from agro-industrial by-products—such as sugar beet molasses—exemplifies circular economy principles and represents an opportunity to reduce food system waste while enhancing feed and food quality. This aligns with global objectives for more sustainable and resilient agri-food chains. Given its multifunctionality, betaine could be considered a model compound for the development of next-generation functional additives capable of responding to both nutritional and environmental challenges. Looking forward, targeted research should focus on (i) the standardization of supplementation protocols for different species and production goals, (ii) the synergistic effects of betaine with other dietary interventions, (iii) its role in precision nutrition strategies tailored to individual metabolic profiles, and (iv) its integration in climate-adaptive livestock and aquaculture systems. Particularly in regions such as the Mediterranean, where environmental stress is expected to intensify, the strategic inclusion of betaine in nutritional programs may play a key role in preserving animal health and productivity.

In conclusion, betaine is not merely a feed additive but a metabolic modulator with wide applicability in health promotion and sustainable production. Its future use will benefit from multidisciplinary approaches combining animal nutrition, molecular biology, food technology, and environmental science to fully exploit its potential for the benefit of both human and animal populations.

## Figures and Tables

**Table 3:** Effects of betaine supplementation in cattle.

Source	Dosage	Breed and Category	Diet Type	Main Effects ^1^	Reference
Feed-grade betaine	10.5, 21 g/d	Not reported, steers	Steam-flaked and dry-rolled corn	FI, D%, CC	[200]
Feed-grade betaine	10.5, 21 g/d	Various breed, steers	Steam-flaked and dry-rolled corn	DMI, D%, CC	[184]
Feed-grade betaine	20 g/d	Aberdeen Angus, steers	50:50 rolled corn:rolled milo	ADG, FT	[201]
Feed-grade betaine	0.25 g/kg of BW	Zebu, steers	Not reported	TrS	[202]
Rumen-protected betaine	45 g/d	Holstein, lactating cows	Corn silage-based TMR ^2^	STC; LDL	[203]
Anhydrous betaine	50, 100, 150 g/d	Holstein, lactating cows	Corn silage-based TMR	MY, FCM, %F, RpH, TrVFA, A:Pr; Nd; NEFA, BHB	[204]
Rumen-unprotected betaine	25, 50, 100 g/d	Holstein, lactating cows	Triticale silage-based TMR	MY; %P	[205]
Feed-grade betaine	10, 15, 20 g/d	Holstein, lactating cows	Corn silage-based TMR	FI, MY; %L; %P, T-AOC, MDA, SOD, CPK	[206]
Feed-grade betaine	57 and 114 mg/kg BW	Holstein, lactating cows	Hay-based TMR	DMI, MY, BGl	[189]
Betaine-containing molasses	1.1 and 1.4 kg DM/d	Holstein, dry-off cows	Corn silage-based TMR	MY, %F, FCM, ECM	[207]
Natural betaine	2 g/kg DM	Holstein, lactating cows	Pasture with concentrate	MY, MPY, MFY, MF, CI, Ru	[185]
Feed-grade betaine	50 g/d	Karan Fries, lactating cows	Concentrate mixture and roughages	NEFA, Cortisol, FCy	[208]
Feed-grade betaine	20 g/d	Holstein, dry-off cows and newborn calves	Corn silage-based TMR	BTP, Gc	[199]
Feed-grade betaine	4.0 g/kg DM	Holstein, lactating cows	Corn silage-based TMR	MY, FCM, %F, Fe, Nd, TrVFA, NH_3_-N, Rmea, BGl,	[190]
Feed-grade betaine	15 and 30 g/d	Holstein, lactating cows	Corn silage-based TMR	MY, SCC, TrVFA, MCP; NH_3_-N, Nd, T-AOC, MDA, GSH-Px, SOD	[209]
Rumen-protected betaine	20 g/d	Holstein, lactating cows	Corn silage-based TMR	DMI, MY, %P, %F, %L, SCC, NEFA, PT, BGl, BUN,	[210]
Betaine hydrochloride	30 g/d	Aberdeen Angus cows	Concentrate mixture and wheat straw	HGB, RBC, RT, PR	[211]
Feed-grade betaine	80 g/d	Holstein, lactating cows	Corn silage-based TMR	Urea, ALT, SOD, BGl, MY, FCM, %F, DMI	[196]

^1^ %F: Milk fat; %L: Milk lactose; %P: Milk protein; A:Pr: acetate to propionate rate; ADG: average daily body weight gain; ALT: alanine aminotransferase; BGl: Blood glucose; BHB: b-hydroxybutyrate; BTP: Blood total protein; BUN: blood urea nitrogen; CC: carcass characteristics; CI: Concentrate intake; CPK: creatine phosphate kinase; D%: Dressing %; DMI: Dry Matter Intake; ECM: Energy Corrected Milk; FCM: fat corrected milk; FCy: follicular cyst; Fe: Feed efficiency; FI: Feed intake; FT: Fat thickness; Gc: Globulin concentrations; GSH-Px: glutathione peroxidase; HGB: hemoglobin concentration; LDL: Low-density lipoproteins; MCP: microbial protein; MDA: malondialdehyde; MF: Milking frequency; MFY: Milk fat yield; MPY: Milk protein yield; MY: Milk yield; Nd: nutrient digestibility; NEFA: non-esterified fatty acids; NH_3_-N: ammonia; PR: Pulse rate; PT: Plasma triglyceride; RBC: red blood cell; Rmea: ruminal microbial enzyme activity; RpH: ruminal pH; RT: Rectal temperature; Ru: Rumination; SCC: somatic cell count; SOD: superoxide dismutase activity; STC: Serum total cholesterol; T-AOC: total antioxidant capacity; TrS: transport stress; TrVFA: Total ruminal volatile fatty acid. ^2^ TMR: Total mixed ration.

## Data Availability

The supplementary data are available from the corresponding author upon reasonable request.

## Abbreviations

SMB	Simulated moving bed
ALD	Alcoholic fatty liver disease
MAFLD	Metabolism-associated fatty liver disease
ADH	Alcohol dehydrogenase
MEOS	Microsomal ethanol-oxidizing system
Met	Methionine
SAM	S-adenosylmethionine
SAH	S-adenosylhomocysteine
FFAs	Free fatty acids
VLDL	Very low-density lipoproteins
NOS	Nitric oxide synthase
BHMT	Homocysteine methyl transferase
ADG	Average daily gain
FCR	Feed conversion ratio
MAT	Methionine adenosyltransferase
PUFAs	Polyunsaturated fatty acid
ALA	Alpha-linolenic acid
EPA	Eicosapentaenoic acid
DHA	Docosahexaenoic acid
CKD	Chronic kidney disease
FASS	Fatty acid synthase
ACC	Acetyl-CoA carboxylase
